# Enhanced Encapsulation of Linalyl Acetate in Cyclodextrin-Based Metal–Organic Frameworks for Improved Stability

**DOI:** 10.3390/molecules30132698

**Published:** 2025-06-23

**Authors:** Cheng Zhang, Lirong Zhang, Meiting Zhao, Ning Shao, Shuo Song, Xiaolan Zhu

**Affiliations:** 1 Institute of Advanced Technology, University of Science and Technology of China, Hefei 230031, China; zhangcheng123@mail.ustc.edu.cn; 2 Research Center of Tobacco and Healthy, University of Science and Technology of China, Hefei 230086, China; songshuo@mail.ustc.edu.cn; 3 Center of Technology, Anhui Jiaotianxiang Biological Technology Corporation, Xuancheng 242099, China; zzhaomeit@163.com (M.Z.); nshao@mail.ustc.edu.cn (N.S.); 4 Anhui Provincial Key Laboratory of Aerosol Analysis, Regulation and Biological Effect, Institute of Advanced Technology, University of Science and Technology of China, Hefei 230031, China

**Keywords:** linalyl acetate, metal–organic frameworks, encapsulation, cyclodextrin, inclusion complex

## Abstract

Linalyl acetate (LA), a key volatile component in essential oils, is extensively utilized in fragrance, food, and cosmetic industries. Nevertheless, its practical applications are constrained by rapid evaporation and physicochemical instability. This study developed novel cyclodextrin–metal–organic frameworks (CD-MOFs) crystallized from β-cyclodextrin (β-CD) and γ-cyclodextrin (γ-CD) with potassium hydroxide, demonstrating superior structural properties for LA encapsulation. Through comparative analysis with native CDs, the synthesized CD-MOFs exhibited highly ordered crystalline architectures and uniform morphological characteristics. The LA encapsulation capacity of the γ-CD-MOF was systematically evaluated under different conditions using a three-level factorial design via RSM. Optimization revealed maximum encapsulation efficiency (25.9%) under ideal conditions—an LA:γ-CD-MOF mass ratio of 3.8:1, 60.9 °C incubation temperature, and 49.3 min processing time—representing a 2.39-fold enhancement over conventional CD encapsulation. Thermal stability analysis demonstrated remarkable improvement, with LA-γ-CD-MOF complexes showing an onset decomposition temperature of 215 °C, 135 °C higher than that of free LA. Compared with LA-γ-CD, LA coated with γ-CD-MOFs still retained 55.7% at 80 °C for 75 min, with the release rate reduced by about 45.3%. These findings establish the potential of γ-CD-MOFs as effective carriers for thermolabile and volatile compounds in functional food and cosmetic industries.

## 1. Introduction

Linalyl acetate (LA), a naturally occurring monoterpene ester, serves as a principal aromatic constituent in essential oils derived from lavender, bergamot, and clary sage [1]. Renowned for its complex olfactory profile combining floral, fruity, and herbal notes, LA has established itself as an indispensable ingredient in fragrance, flavor, and cosmetic formulations [2]. Beyond its sensory attributes, LA demonstrates significant therapeutic potential, exhibiting anti-inflammatory, antimicrobial, and anxiolytic properties [3]. In food applications, LA contributes a distinctive citrusy-sweet flavor profile to confectionery, baked goods, and beverages, enhancing product palatability and consumer acceptance [4]. However, the industrial utilization of this compound is greatly limited by its physicochemical properties, including volatility, oxidative susceptibility, and thermal instability [5]. Perhaps encapsulation of LA is a good way to change this situation.

**Figure 1:**
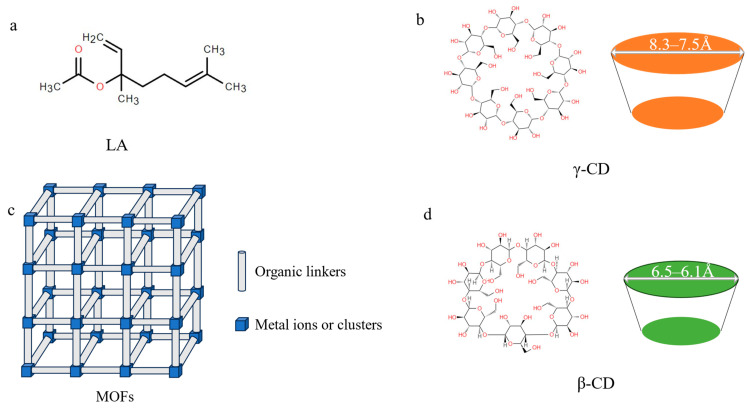
Structural diagram of LA (**a**), γ-CD (**b**), MOFs (**c**), and β-CD (**d**).

The quest for effective encapsulation systems has driven significant advancements in material science, particularly for volatile compound stabilization. While traditional porous materials such as activated carbon, carbon nanotubes, and zeolites have been widely employed in catalysis [6], drug delivery [7], and environmental remediation [8], these applications are often constrained by limited efficiency, pore blockage, and stringent operational requirements [9]. The structures of CDs are shown in Figure 1. Cyclodextrins (CDs) are cyclic oligosaccharides composed of α-1,4-linked glucose units, forming a truncated cone structure with a hydrophobic cavity and hydrophilic exterior [10]. This unique architecture enables them to encapsulate volatile compounds via host–guest interactions, stabilizing these molecules by reducing their volatility and extending their applications in food, pharmaceuticals, and cosmetics [11,12,13]. Subsequently, cyclodextrin–metal–organic frameworks (CD-MOFs) have emerged as a revolutionary class of hybrid materials, combining the molecular recognition capabilities of cyclodextrins with the structural versatility of MOFs [14]. The encapsulation of volatile compounds within CD-MOFs offers several advantages, including enhanced loading capacity, improved physicochemical stability, and controlled release properties [15]. The porous structure of CD-MOFs provides a high surface area and tunable pore sizes, which facilitate the efficient entrapment of volatile molecules [16]. This not only prevents the premature evaporation of the encapsulated compounds but also protects them from degradation due to environmental factors such as light, heat, and oxygen. Moreover, the controlled release properties of CD-MOFs are particularly beneficial for applications requiring sustained and targeted delivery of volatile compounds. This makes CD-MOFs an attractive option for the development of advanced delivery systems in the food and flavor industries [17]. However, the direct application of MOFs in the food industry has been restricted because of their toxicity, non-food-grade organic linkers, and the use of transition metal ions [18,19].

Fortunately, the development of food-compatible CD-MOFs, synthesized through vapor diffusion methods using alkali metal ions and cyclodextrins, represents a paradigm shift in bioactive compound encapsulation [20]. These crystalline frameworks exhibit exceptional molecular recognition capabilities, tunable porosity, and harmless property, making them ideal for food and pharmaceutical applications [21,22,23,24]. The unique structural features of CD-MOFs, including their well-defined channels and cavities, enable precise molecular encapsulation and protection of sensitive bioactive compounds. For hydrophobic bioactive molecules, γ-CD-MOF has shown exceptional loading capacities [25]. For example, curcumin encapsulation achieved 82.82% loading efficiency and dramatically increased curcumin solubility [26]. Similarly, the encapsulation efficiency of resveratrol in γ-CD-MOF could reach 66.5%, and the encapsulation efficiency could even reach 91.3% after modification of γ-CD-MOF [27]. Compared with free carvacrol, carvacrol coated with β-CD-MOF only released 4.9% by day 30, and the release rate was reduced by about 15% [28]. Nana et al.’s development of micro- and nanoscale alkaline CD-MOFs significantly improved sucralose’s thermal stability, maintaining 92% stability after heat treatment at 120 °C for 2 h [29]. Recent advancements have further expanded CD-MOF applications: menthol encapsulation reached 27.1% loading capacity with 30.6% efficiency [30], while catechin stabilization in nanoscale CD-MOFs improved light stability 3.2-fold and thermal stability 2.8-fold [19]. Moreover, the incorporation of polyethylene glycol 10000 (PEG10000) as a regulator during the preparation process could enhance the embedding rate of nanoscale CD-MOFs for bioactive molecules: the bioavailability of cyclosporine A encapsulation increased by 3.5 [31].

To further expand the application of LA in food and flavor, this study aims to systematically investigate the encapsulation and release properties of LA in CD-MOFs, employing a dual-synthesis approach: ultrasound-assisted synthesis for γ-CD-MOF and vapor diffusion for β-CD-MOF. Through comprehensive optimization of temperature, duration, and mass ratio parameters, we establish optimal encapsulation conditions. Furthermore, this work provides detailed characterization of the inclusion complex’s stability and controlled release properties, addressing critical gaps in volatile compound stabilization for food and cosmetic applications.

## 2. Results and Discussion

### 2.1. Structural Characterization of CD-MOFs

#### 2.1.1. Crystal Morphology

**Figure 2:**
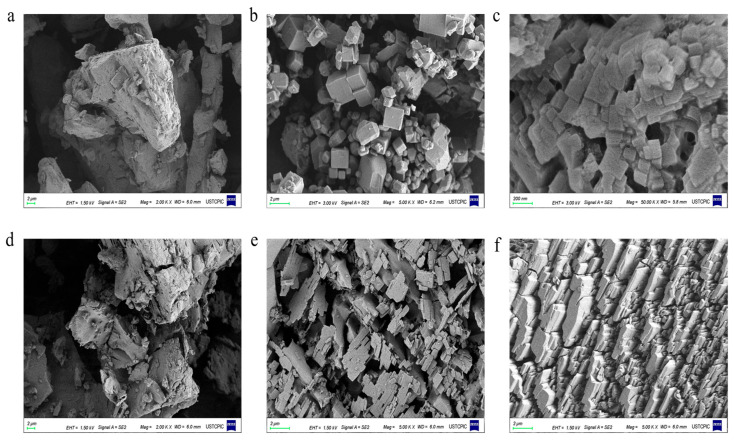
SEM images of γ-CD (**a**), γ-CD-MOF (**b**), LA-γ-CD-MOF (**c**), β-CD (**d**), β-CD-MOF (**e**), and LA-β-CD-MOF (**f**).

The morphological characteristics of cyclodextrin and its metal–organic framework materials (CD-MOFs) are compared and presented in Figure 2. It can be found that both γ-CD (Figure 2a) and β-CD (Figure 2d) particles exhibited irregular morphology features, with a wide range of particle size distribution and disordered stacking. In contrast, both γ-CD-MOFs (Figure 2b) and β-CD-MOFs (Figure 2e) exhibited highly ordered crystalline structures, which resulted in a significant reduction in crystal particle size. The primary distinction lies in their morphological differences: β-CD-MOFs formed rectangular crystals, whereas γ-CD-MOFs adopted a cubic crystal, which was consistent with the results reported in the literature [32,33].

#### 2.1.2. XRD Analysis

**Figure 3:**
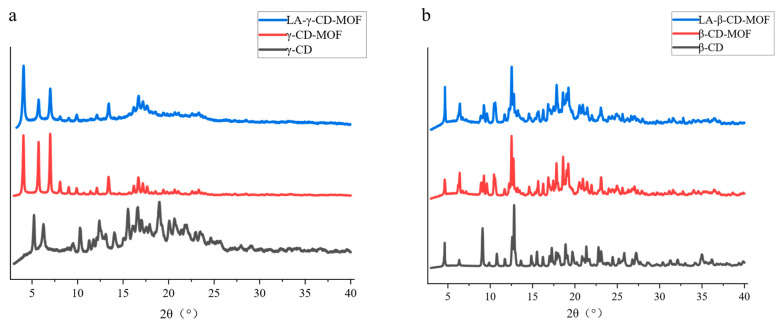
X-ray diffraction pattern of CD and its derivatives: (**a**) γ-CD and (**b**) β-CD.

Figure 3 presents a comparison of the XRD patterns of the native CDs and CD-MOFs. Obviously, the XRD characteristics of CD-MOFs are significantly different from those of the corresponding CDs. γ-CD-MOF (Figure 3a) exhibited typical Bragg diffraction peaks at diffraction angles of 4.0°, 5.6°, 6.9°, 13.4°, and 16.6°, while those of β-CD-MOF appeared at 4.7°, 6.4°, 9.1°, 12.7°, and 18.7° (Figure 3b). The results indicate that the coordination effect of potassium ions effectively induced cyclodextrin molecules to form more ordered crystal structures. This structural reorganization process is not only reflected in the peak evolution of XRD patterns, but is also consistent with the regular crystal morphology displayed by SEM.

### 2.2. Optimization of Encapsulation Efficiency

The ratio of target molecules to the profiled material and the conditions of inclusion (such as temperature and time) often have a crucial impact on the encapsulation efficiency of CD-MOFs [34]. Therefore, the effects of incubating temperature (A), incubating time (B), and mass ratio m_LA_:m_CD-MOF_ (C) on the percentage of LA content (% LAC, Y) in the inclusion complex were investigated. Due to the poor water solubility of β-CD-MOF, it was unsuitable for subsequent experiments and applications. So, water-soluble γ-CD-MOF was selected for subsequent encapsulation experiments. To further study the interactive influence of these factors, a three-factor, three-level Box–Behnken trial design was used in the response surface test, and the percentage of LAC was selected as the response value. The experimental factors and levels are shown in Table 1.

Through multiple linear regression and binomial fitting analysis of the experimental results in Table 2, the regression equation for the content of LA was obtained as Y = −11.85 + 0.64A + 0.09B + 8.43C + 0.0001AB + 0.009AC − 0.004BC − 0.005A^2^ − 0.0005B^2^ − 1.24C^2^. Variance analysis was performed on the regression equation, and the results are shown in Table 2. According to the results of the ANOVA, the F value of the regression model is 198.00 (*p* < 0.01), and the significance level is much less than 0.0001, indicating that the regression variance model is highly significant. Meanwhile, the misfit error is not significant, indicating the reliability of the model. Among the three factors, m_LA_:m_CD-MOF_ had a very significant effect on the content of LA (*p* < 0.0001), followed by the reaction time (*p* = 0.0044). In addition, the quadratic terms A2 and C2 also had significant effects on Y. The regression coefficient R^2^ = 0.9961 (>0.85) indicates that the equation has a good fit. Therefore, the experiment design is reliable, and it can be used to analyze and predict the LAC in the inclusion complex.

**Figure 4:**
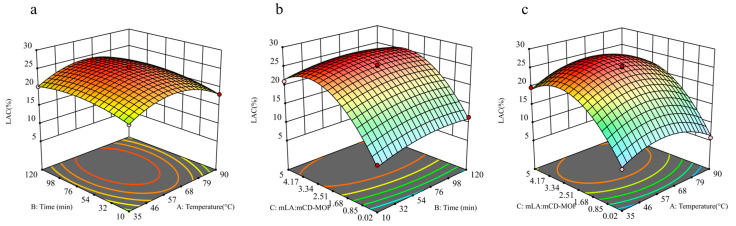
Response surface plot of effect of interaction of various factors on LAC. The effect of the interaction between Temperature and Time (**a**), Time and m_LA_:m_CD-MOF_ (**b**), m_LA_:m_CD-MOF_ and Temperature (**c**) on the percentage of LA content.

According to the results of the analysis of variance, the response surface analysis 3D plot is shown in Figure 4. It can be seen that the effect of the interaction between incubating temperature (A) and incubating time (B) on the percentage of LA content was not significant, but the interactions between m_LA_:m_CD-MOF_ (C) with both of them had a significant effect on the LAC. This is consistent with the data in Table 2, where the *p*-value for AB (0.5618) is much higher than the *p*-values for AC (0.0958) and BC (0.1467). As shown in Figure 4, with the increase in incubating temperature, the percentage of LAC initially increased and then decreased to a small extent. When incubating time was fixed, the percentage of LAC increased first and then decreased with the increase in temperature and m_LA_:m_CD-MOF_. When m_LA_:m_CD-MOF_ was between 3.0 and 4.5, the percentage of LA content reached a high level (Figure 4b). This indicates that at a low m_LA_:m_CD-MOF_ value, an elevated m_LA_:m_CD-MOF_ promotes the entry of LA in the pores of the γ-CD-MOF material to form the structure of host and guest and increases the amount of LA embedded. With the ever-increasing amount of LA, the adsorption of LA gradually reached saturation due to limited adsorption sites provided by CD-MOFs. And when the m_LA_:m_CD-MOF_ value was above 4, the percentage of LAC decreased slightly. From Figure 4a, it can be seen that when m_LA_:m_CD-MOF_ was immobile, the percentage of LAC initially increased and then decreased slightly with the increase in incubating temperature and time. An appropriate temperature could accelerate the encapsulation process and promote intermolecular interactions between LA and γ-CD-MOFs. But excessive temperature may results in the collapse of the crystal structure of γ-CD-MOFs or molecular leakage. On the other hand, sufficient encapsulation time could ensure that molecules diffused sufficiently and were stable in the pore structure of γ-CD-MOFs, while an excessively long encapsulation time could lead to weakened intermolecular interactions, thereby reducing encapsulation efficiency.

Table 2 presents the results of the analysis of variance (ANOVA) based on the percentage of LAC in the experimental design. The provided data can accurately analyze the impact of each parameter on the LAC. By analyzing the data, the optimal experimental conditions can be obtained: temperature: 60.9 °C; time: 49.3 min; m_LA_:m_CD-MOF_: 3.8. Three repeated experiments were carried out under the above conditions, and the average percentage of LAC was 25.9, which is basically consistent with the predicted value. This indicates that the pyrolysis condition parameters are reliable. Under the same conditions, the average percentage of LAC in γ-CD and β-CD-MOF is 10.84 and 15.23, respectively, significantly lower than that in the γ-CD-MOF inclusion complex.

### 2.3. Characterization of LA-γ-CD-MOF and Intermolecular Interaction

The morphological characteristics of LA-γ-CD-MOF are presented in Figure 2c. It can be seen that LA-γ-CD-MOF has similar highly ordered crystalline structures to γ-CD-MOF, with the exception of the cubic crystal slightly increasing in particle size and having a slightly rougher surface due to the incubation of LA. The morphological characteristics of LA-β-CD-MOF are very similar, except for the rectangular crystal. On the other hand, Figure 3 also presents the XRD patterns of LA-γ-CD-MOF. It is worth noting that LA-γ-CD-MOF maintained a diffraction peak position similar to that of γ-CD-MOF [35], which could be attributed to the fact that the introduction of amorphous LA did not significantly interfere with the regular crystal structure of CD-MOF. This demonstrates that LA completely entered the pores of γ-CD-MOF, and the formation of host–guest interactions between LA and γ-CD-MOF did not affect the crystal structure of CD-MOF, thus not triggering the generation of new characteristic peaks.

**Figure 5:**
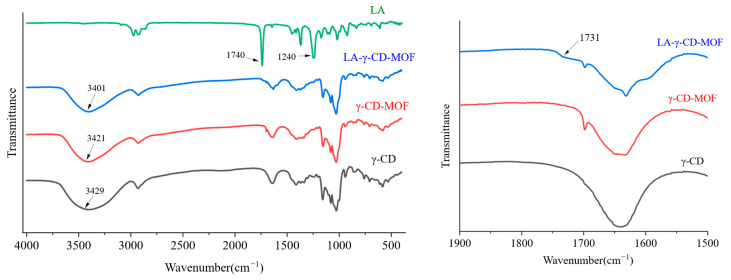
FTIR of LA, γ-CD-MOF, and LA-γ-CD-MOF.

To further verify the interaction between LA and γ-CD-MOF, FTIR analysis of several materials was performed. The results (in Figure 5) show that the spectrum of LA-γ-CD-MOF is significantly different from that of pure LA but similar to that of γ-CD-MOF. Pure LA has a strong and sharp absorption peak at approximately 1740 cm⁻^1^, which is attributed to the carbonyl (C=O) characteristic peak in the ester group, exhibiting high intensity and sharpness. In addition, a moderate intensity absorption peak is observed at approximately 1240 cm⁻^1^, attributed to the oxygen bridge vibration in the ester bond (R-O-CO-R). However, the peak on the spectrum of LA-γ-CD-MOF is weakened and the peak at about 1740 cm⁻^1^ is shifted slightly towards a shorter wavelength (1731 cm⁻^1^). On the other hand, the broad characteristic absorption peak at 3700–3000 cm−1, assigned to the stretching vibration of the –OH groups from γ-CD (3429 cm⁻^1^), is shifted slightly towards a shorter wavelength (3421 cm⁻^1^) in γ-CD-MOF and further shifted slightly towards a shorter wavelength (3401 cm⁻^1^) in LA-γ-CD-MOF. This result suggests the presence of hydrogen bonding interactions between LA and γ-CD-MOFs. The FTIR results indicate that LA was successfully encapsulated in γ-CD-MOF and formed a non-covalent host–guest inclusion complex. This is consistent with the results of previous studies [36].

### 2.4. Thermal Stability Analysis of LA, γ-CD, and Their Inclusion Complex

**Figure 6:**
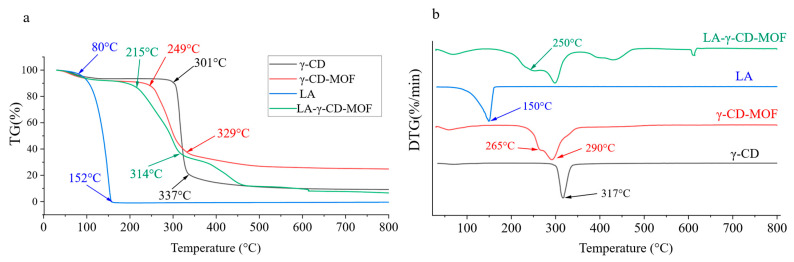
TGA (**a**) and DTG (**b**) of γ-CD, γ-CD-MOF, and LA-γ-CD-MOF.

The thermogravimetric analysis curve in Figure 6a reveals the differences in thermal stability behavior of γ-CD and its derivatives. As shown in Figure 6, the weight of LA rapidly decreased in the range of 80–152 °C and the decomposition rate reached over 98% at 150 °C, indicating the low thermal stability of LA molecules. By comparison, only a 10% mass loss occurred in LA-γ-CD-MOF at 200 °C, confirming that the encapsulation process made LA molecules enter the metal–organic framework of γ-CD-MOF and enhance its thermal stability. The thermogravimetric curve shows that the initial weight loss stage (about 10%) at 40–100 °C was due to the volatilization of residual solvents. The thermal degradation processes of γ-CD and its derivatives are reflected in a higher temperature range. Compared with the temperature of the main decomposition of natural γ-CD (301 °C), that of γ-CD-MOF and LA-γ-CD-MOF is significantly shifted to a lower temperature range, 200–350 °C, which is consistent with the thermal behavior of cyclodextrin-based materials reported by previous researchers [37]. This difference in thermal stability of materials might be due to two factors: Firstly, the strength of metal–organic coordination bonds was weaker than the intermolecular forces between natural CD molecules. Secondly, the porous skeleton structure increased the specific surface area and accelerated the heat conduction process.

To further elucidate the interaction between LA and γ-CD-MOF, DTG analysis was used for characterization (Figure 6b). The results show that pure LA exhibited a significant endothermic peak at 150 °C. Meanwhile, γ-CD-MOF exhibited two distinct endothermic peaks at 265 °C and 290 °C. In the LA-γ-CD-MOF composite material, the endothermic peak originally located at 265 °C shifted to a lower temperature (250 °C), and the peak shape became wider, indicating that LA was successfully encapsulated in γ-CD-MOF and decreased the thermal stability of γ-CD-MOF.

### 2.5. Release Behaviors of Combined LA in LA-γ-CD-MOF

**Figure 7:**
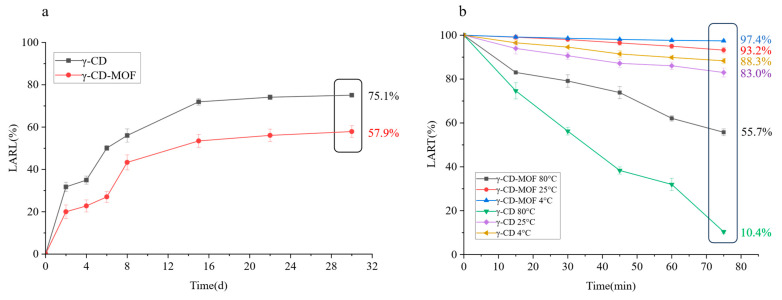
LA release rate of γ-CD-MOF and γ-CD during storage at 25 °C for 30 d (**a**), and influence of time and temperature on LA retention of γ-CD-MOFs and γ-CDs (**b**).

In this study, inclusion complexes (ICs) were prepared under high pressure at 60.9 °C; high pressure facilitates interfacial interactions between LA molecules and the γ-CD-MOFs, and the correct orientation of LA molecules for diffusion into the pores of the crystal structure. The main driving forces of IC formation are the large internal surface area of the pores and hydrophobic interactions. Compared with γ-CDs, γ-CD-MOFs possess a much more ordered structure (Figure 2a), greater crystallinity (Figure 3), and a microporous structure with a large internal surface area, which greatly promoted the entry of LA in the pores of the γ-CD-MOF material to form the structure of host and guest and enhanced the stability of LA. To further investigate the stability of LA after encapsulation, the percentage of LA in LA-γ-CD-MOFs and LA-γ-CD under different conditions was compared. Figure 7 presents the evolution of %LAC with time (0–30 d) and temperature (4 °C, 25 °C, and 80 °C). From Figure 7a, it can be seen that the release curves demonstrate two stages: a rapid growth stage and a plateau stage. Firstly, from 0 to 4–5 days, those redundant LA molecules encapsulated in γ-CD-MOF under thermal treatment could not find enough pores in the γ-CD-MOF material and therefore were released rapidly from the LA-γ-CD-MOF inclusion complex. Subsequently, from 5–8 days till 30 days, all the remaining LA entered the pores of the γ-CD-MOF material to form the stable non-covalent host–guest structure, and the release rate of LA was stable (plateau stage). After 30 days, the release rate of LA in LA-γ-CD-MOF was around 57.9%, while in LA-γ-CD, the release rate of LA was as high as 75.1%. This indicates that γ-CD-MOFs are superior to γ-CD in both capacity and persistence of encapsulation. The high volatility of LA often limits its potential application in food, and encapsulation in γ-CD-MOFs could delay the release of LA to improve the overall aroma and quality of food products during production and storage.

Temperature was another significant factor which indirectly influenced the binding sites between LA molecules and the profiled materials. From Figure 7b, it can be seen that the LA retention percentage in the LA-IC samples significantly decreased upon increasing the temperature from 4 to 80 °C. Compared with LA-γ-CD, LA coated with γ-CD-MOFs still retained 55.7% at 80 °C for 75 min, with the release rate reduced by about 45.3%. It can be seen that, under the same conditions, the retention rate of LA-γ-CD-MOF was significantly higher than that of LA-γ-CD. The increase in temperature led to an accelerated release of LA encapsulated in ICs, which can be explained according to the Arrhenius equation:

$$k=Ae^{-\frac{E_{a}}{RT}}$$

The reaction rate constant (*k*) increases exponentially as temperature (*T*) rises. The influence of *T* on *k* is non-linear and is dominated by the activation energy (*Ea*). For a specific thermal release reaction between LA and γ-CD-MOF, *Ea* is a fixed constant value, so k is mainly influenced by *T*. An increase in temperature significantly enhances the kinetic energy of the molecule [38]. The encapsulated LA molecules are more likely to overcome van der Waals forces or hydrogen bonding interactions with γ-CD-MOF at high temperature, thereby accelerating the diffusion rate from the porous framework. Similar studies showed that for every 10 °C increase in temperature, the diffusion rate of guest molecules could be increased 1.5–2 times [39]. The interactions formed between LA and γ-CD-MOF or γ-CD became weaker at higher temperature, thus facilitating the escape of LA from the ICs. Based on the above results, it can be further concluded that γ-CD-MOF can retain a large amount of LA in its pores, reducing its loss during food processing or storage.

## 3. Materials and Methods

### 3.1. Materials

Linalyl acetate (purity ≥ 98%), γ-CD (purity ≥ 98%), and β-CD (purity ≥ 98%) were purchased from Macklin Co., Ltd. (Shanghai, China). Cetyltrimethylammonium bromide (CTAB) was produced by Aladdin Reagent Co., Ltd. (Shanghai, China). Methyl alcohol, potassium hydroxide, anhydrous ethanol, and dichloromethane were from purchased from Sinopharm Chemical Reagent Co., Ltd. (Shanghai, China). All other chemical reagents used in this work were of analytical grade.

### 3.2. Preparation of CD-MOFs

**Figure 8:**
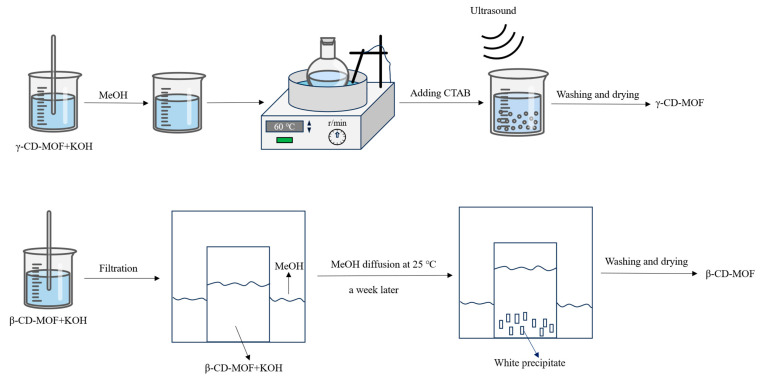
The preparation procedures of γ-CD-MOF and β-CD-MOF.

The preparation of γ-CD-MOF was conducted according to previous ultrasound-assisted methods with some modifications [16,40]. The specific operation process is shown in Figure 8 and described as follows: γ-CD (648 mg, 0.5 mmol) and potassium hydroxide (256 mg, 4.56 mmol) were added into a beaker with 20 mL deionized water and stirred at room temperature. After full dissolution, the solution was filtered through a 0.45 μm filter membrane and the filtrate was transferred to a centrifuge tube containing 12 mL methanol to form a milky white solution. The centrifuge tube was placed in a water bath at 60 °C, and a clear and transparent solution was obtained after standing for 10 min. Then, CTAB (160 mg) was rapidly added into the solution, and an ultrasonic reaction was performed at a frequency of 40 kHz and a power of 600 W for 10 min to obtain the crude product. The crude product was transferred to a beaker and left for 1 h. The precipitate was centrifuged at 10,000 rpm for 10 min and rinsed with methanol 3 times. The centrifuge-separated product was put into a vacuum drying oven, dried at 50 °C for 12 h under vacuum conditions, and cooled to room temperature to obtain γ-CD-MOF with approximately 85% of the yield.

The preparation of β-CD-MOF was carried out according to a previous steam diffusion method [41,42] with some modifications. The specific operation process is shown in Figure 8 and described as follows: β-CD (1.1349 g) and potassium hydroxide (0.4488 g) were added into a beaker with 20 mL deionized water and stirred at room temperature. After full dissolution, the solution was filtered through a 0.45 μm filter membrane and the filtrate was put into a large beaker with 110 mL of methanol. The mixture was sealed with cling film for one week to obtain a white precipitate. Then, the precipitate was centrifuged at 10,000 rpm for 10 min and washed with methanol centrifuge 3 times. After centrifugation, the product was put into a vacuum drying oven, dried at 50 °C for 12 h under vacuum condition, and cooled to room temperature to obtain β-CD-MOF with approximately 75% of the yield.

### 3.3. Encapsulation of LA

The encapsulation of LA was performed as described previously [43], with minor modifications. A certain amount of γ-CD-MOF or β-CD-MOF was added to LA dissolved in ethanol and then hydrothermally treated under certain conditions. A three-level factorial design via a response surface methodology (RSM) experiment was used to investigate the effects of the mass ratio of LA:γ-CD-MOF (1:50, 5:2, and 5:1), temperature (35 °C, 62.5 °C, and 90 °C), and time (10 min, 65 min, and 120 min) on drug loading. The RSM experiment was designed and analyzed via SPSS 21.0 software (SPSS, Inc., Chicago, IL, USA). After heating, the mixture was washed with ethanol three times to remove uncoordinated LA, and finally vacuum-dried at room temperature for 12 h to obtain the inclusion complex. The inclusion complex of γ-CD-MOF loaded with LA was referred to as LA-γ-CD-MOF, and the inclusion complex of β-CD-MOF loaded with LA was referred to as LA-β-CD-MOF.

### 3.4. Characterization of CD-MOFs and LA-CD-MOFs

All of the CD-MOF and LA-CD-MOF samples were ground and passed through a sieve of 200 mesh. The X-ray diffraction (XRD) pattern of the crystal was recorded using an X-ray diffractometer (TTR III, Rigaku, Tokyo, Japan) under 44 kV and 27 mA Cu-K α rays (λ = 1.54 nm). The analytical diffraction pattern was obtained in the range of 3° to 40° (2θ) at a scanning speed of 5 °/min and a step size of 0.01°. The surface morphology of CD-MOF and LA-CD-MOF particles was observed by using a scanning electron microscope (Gemini SEM 500, Hitachi, Tokyo, Japan).

The FTIR spectra of LA, CD, CD-MOFs, and LA-CD-MOFs were obtained using an FTIR spectrometer (Nicolet iN10, Ettlingen, Germany). Each sample was pressed into a KBr disk and then scanned between 4000 cm⁻^1^ and 400 cm⁻^1^. The spectrum was obtained at a resolution of 4 cm⁻^1^ with 64 scans against an air background. The FTIR analysis is automatically baseline-corrected by OMNIC 8.0 software. Thermal gravimetric analysis (TGA, 209F1, PerkinElmer, Waltham, MA, USA) was adopted to analyze the thermostability of LA, γ-CD-MOFs, and LA-γ-CD-MOFs. Each sample was heated from 30 °C to 800 °C in an air atmosphere at a speed of 10 °C/min for testing.

### 3.5. Quantitative Determination of LA Content in Inclusion Complex

A total of 30 mg of dried solid inclusion complex samples (including LA-CDs, LA-CD-MOFs) was admixed with 5 mL of distilled water and stirred for 30 min. After complete dissolution, about 1mL dichloromethane was used to extract LA from the solution, and the extraction process was repeated 3 times. The supernatant was combined and the volume was set to 10 mL. The content of LA was tested using UV-VIS spectrophotometry (TU-1901, Persee, Shanghai, China) [44]. The instrument parameters were as follows: UV wavelength scanning range: 200–600 nm; bandwidth: 20 nm; sampling interval: up to 0.5 nm; medium speed scanning; and LA detection wavelength: 227 nm. The percentages of LA content (% LAC), LA retention (%LART), and LA release (% LARL) were calculated by using Equations (1)–(3) enumerated below:

(1) $$\%\;LAC=\frac{Extracted\;LA}{Weight\;of\;coplexation\;powder\;}\times 100\%,$$

(2) $$\%\;LART=\frac{LAC_{t,T}}{LAC_{inj}\;}\times 100\%,$$

(3) $$\;\%\;LARL=\frac{LAC_{inj}-LAC_{t,T}}{LAC_{inj}\;}\times 100\%,$$

where *LAC_inj_* and *LAC_t,T_* express the % LAC retained in the inclusion complex at the beginning and after t (min) or T (°C). The results were recorded as the mean ± standard deviation of three measurements.

## 4. Conclusions

This study has incorporated LA into γ-CD-MOFs, resulting in improved loading capacity, thermal stability, and controlled release of LA compared with individual γ-CD encapsulation. The characterization results confirm that LA was successfully encapsulated into γ-CD-MOF and occupied the highly ordered crystalline porous structures of γ-CD-MOFs via hydrogen bonding interactions. The encapsulation conditions of LA were optimized using the RSM design to obtain the highest percentage of LA in γ-CD-MOFs, with the following parameters: temp: 60.9 °C; time: 49.3 min; m_LA_:m_CD-MOF_: 3.8. Under these conditions, the average percentage of LAC in γ-CD-MOFs was 25.9, which was 2.39 times higher than that in γ-CD ICs. Moreover, the LA-γ-CD-MOFs presented improved thermal stability with a decomposition temperature 135 °C higher than that of free LA. When compared with LA-γ-CD, the release rate of LA coated with γ-CD-MOFs was reduced by about 45.3% at 80 °C for 75 min, which indicates that the γ-CD-MOFs have strong potential as a carrier for volatile compounds or for the encapsulation of aroma compounds.

## Figures and Tables

**Table 1:** Design and conclusion of response surface testing.

Run	A Temperature/°C	B Time/min	C m_LA_:m_CD-MOF_	Y LAC/%
1	35	65	5	20.01
2	35	10	2.51	19.42
3	35	120	2.51	20.23
4	62.5	65	2.51	24.96
5	62.5	10	0.02	8.71
6	62.5	65	2.51	25.21
7	90	65	0.02	10.97
8	90	65	5	20.36
9	35	65	0.02	7.47
10	62.5	65	2.51	24.61
11	62.5	65	2.51	25.48
12	90	10	2.51	19.89
13	62.5	120	0.02	11.21
14	62.5	10	5	21.10
15	90	120	2.51	20.38
16	62.5	120	5	21.83
17	62.5	65	2.51	25.43

**Table 2:** Analysis of variance of regression equations.

Source	Sum of Squares	*df*	Mean Square	*F*-Value	*p*-Value	
Model	675.93	9	75.10	198.00	<0.0001	significant
A-Temperature	0.2888	1	0.2888	0.7614	0.4118	
B-Time	6.48	1	6.48	17.08	0.0044	
C-m_LA_:m_CD-MOF_	307.77	1	307.77	811.39	<0.0001	
AB	0.1406	1	0.1406	0.3707	0.5618	
AC	1.40	1	1.40	3.70	0.0958	
BC	1.01	1	1.01	2.66	0.1467	
A^2^	71.05	1	71.05	187.30	<0.0001	
B^2^	11.57	1	11.57	30.51	0.0009	
C^2^	250.14	1	250.14	659.47	<0.0001	
Residual	2.66	7	0.3793			
Lack of Fit	2.14	3	0.7124	5.50	0.0665	not significant
Pure Error	0.5179	4	0.1295			
Cor Total	678.58	16				
R^2^						0.9961
Adjusted R^2^						0.9911

## Data Availability

The original contributions presented in this study are included in the article; further inquiries can be directed to the corresponding author.
